# Supplementary material for: Novel Linalool-Silver nanoparticles: Synthesis, characterization, and dual approach evaluation via computational docking and antibacterial assays
Source: PLoS One. 2025 Oct 28;20(10):e0333617. doi: 10.1371/journal.pone.0333617 (PMC12561953; doi:10.1371/journal.pone.0333617)
Supplement: Attachment — Submitted filename: Response to Reviewers.docx [file pone.0333617.s002.docx]

**Response Letter to the Reviewer Comments**

We sincerely thank the editor and reviewers for their detailed and constructive feedback on our manuscript. We have carefully addressed all comments and revised the manuscript accordingly. Below, we provide a point-by-point response to each comment.

**Reviewer 1 Comments:**

**Comment 1:** The abstract is overly lengthy and should be made more concise. Please focus primarily on the key results and implications. Avoid extensive background or methodological detail in this section.

**Response:** We acknowledge the reviewer’s comment. The abstract has been revised to focus more on the key findings and their implications while minimizing background and methodological details.

**Comment 2:** The introduction is too brief and lacks depth. It should be expanded to provide a more comprehensive background of the topic. Additionally, please incorporate recent references from 2024 and 2025 to reflect the current state of research and strengthen the rationale of the study.

**Response:** Thank you for your valuable comment. We have expanded the introduction to provide a more comprehensive background by highlighting the role of nanoparticles in pathogen control, the antibacterial potential of AgNPs, and the activity of linalool-rich essential oils. Recent literature from 2024 and 2025 has also been incorporated to reflect current research.

**Comment 3:** The figures provided are of low resolution, and the embedded text is not legible. Please replace all figures with high-resolution versions (minimum 300 DPI) to ensure clarity and readability.

**Response:** We sincerely appreciate the reviewer’s feedback. In response, we have significantly enhanced the quality of all figures using the PACE image optimization tool. Each figure has now been uploaded in high resolution (600 dpi) and in TIFF format, as per standard publication requirements.

**Comment 4:** In my opinion, the Results and Discussion sections should be presented together rather than separately. Integrating these sections will allow for a more cohesive interpretation of the findings and help the reader better understand the significance of the results in context.

**Response:** Thank you for the constructive suggestion. The Results and Discussion sections have been merged into a unified section. This integrated format allows for a more fluid interpretation of the findings and aligns better with the manuscript’s narrative flow**.**

**Comment 5:** A detailed comparison of the present results with previously published studies is necessary and discusses how this work advances the field.

**Response:** We acknowledge the reviewer’s comment. We have now included a comparative discussion in the revised manuscript, highlighting how our findings align with previously published studies. This comparison also emphasizes the novelty and advancement of our work in the context of current antibacterial nanoparticle research**.**

**Comment 6:** The manuscript’s language lacks fluency in certain sections. It requires substantial improvement in grammar, sentence structure, and scientific expression. A professional English editing service is recommended.

**Response:** We sincerely appreciate the reviewer’s feedback. In response, the manuscript's English language has been thoroughly improved using the Paperpal editing tool to ensure clarity and readability**.**

**Comment 7:** The conclusion section is too brief and lacks a comprehensive summary of the study’s key findings, implications, and future directions. The authors are advised to expand this section to clearly highlight the major results, significance of the work, and potential applications.

**Response:** We are grateful for the thoughtful insights. We have expanded the conclusion to provide a more comprehensive summary of the key findings, emphasize the significance of the work, and outline its potential applications and future research directions**.**

**Reviewer 2 Comments:**

**Comment 1:** The particle size distribution is highly polydisperse (ranging from ~9 to 680 nm). This raises concerns about batch uniformity, which affects biological reproducibility.

**Response:** We apologize for the lack of clarity in the manuscript. The wording did not accurately reflect our analysis. We have now clarified that Figure 3a presents size distribution data based on intensity from DLS analysis. Although the DLS intensity distribution showed a dominant peak at ~630 nm, this does not necessarily indicate that most particles were of that size. Due to the R⁶ dependence of light scattering, even a small population of large particles can significantly influence intensity measurements. To address this, we constructed a histogram (Figure 3b) using number-based data derived from the same dataset. This revealed that the highest frequency of particles falls below 100 nm, with an average size of approximately 89 nm calculated using Gaussian fitting and nonlinear curve analysis. Furthermore, the PDI value of 0.29 supports the moderate monodispersity and batch uniformity of the sample, indicating suitability for biological applications.

**Comment 2:** Regarding FT IR spectra-Include complete spectra and assign peaks precisely.

**Response:** Thank you for the observation. We have now included the complete FT-IR spectra in the revised manuscript and have precisely assigned the characteristic peaks. Additionally, we have added the FT-IR spectrum of pure linalool alongside that of LN@AgNPs to enable direct comparison and better highlight the functional groups involved in nanoparticle formation. We have revised the description with precise peak assignments, correlating each observed FT-IR band with specific functional groups present in linalool and their interaction with silver nanoparticles.

**Comment 3:** Regarding Antibacterial Assays-Positive controls (AZM) are included, but no comparison with standard AgNPs was provided.

**Response:** Thank you for your valuable comment. We have revised the manuscript to address this point and now provide a comparison of LN@AgNPs with azithromycin (AZM) as a standard antibacterial reference.

**Comment 4:** Regarding Grammar & Language: The manuscript contains several grammatical and typographical errors. Some examples:

“...make it more potential...” → should be “...makes it more potent...”

“...examined for antibacterial in-silico and in-vitro studies...” → revise for clarity.

**Response:** We sincerely appreciate the reviewer’s feedback. In response, the manuscript's English language has been thoroughly improved using the Paperpal editing tool to ensure clarity and readability.
